# The burden of brain disorders in Norway: an analysis of data from the global burden of disease study 2023

**DOI:** 10.1016/j.lanepe.2026.101857

**Published:** 2026-09-10

**Authors:** Lars Jacob Stovner, Ann Kristin Skrindo Knudsen, Lars Lien, Henrik Peersen, Nils Erik Gilhus

**Affiliations:** a Department of Neuromedicine and Movement Science, NTNU Norwegian University of Science and Technology, Trondheim, Norway; b NorHEAD Norwegian Centre for Headache Research, Trondheim, Norway; c Department for Disease Burden, Norwegian Institute of Public Health, Bergen, Norway; d ROPforsk Innlandet Hospital Trust, Brumunddal, Norway; e Faculty of Social and Health Sciences, University of Inland Norway Elverum, Norway; f The Norwegian Brain Council, Oslo, Norway; g Department of Clinical Medicine, University of Bergen, Bergen, Norway; h Department of Neurology, Haukeland University Hospital, Bergen, Norway; i Centre for Disease Burden, Norwegian Institute of Public Health, Bergen, Norway; j Department in Health Sciences in Gjøvik, Norwegian University of Science and Technology, Trondheim, Norway

**Keywords:** Brain disorders, Disease burden, Incidence, Prevalence, Norway

## Abstract

**Background:**

The term ‘Brain disorders’ encompasses conditions affecting the brain and nervous system and includes neurological, mental, neurosurgical and substance use disorders. No study has previously estimated the combined burden of brain disorders in Norway.

**Methods:**

Data source was the Global Burden of Disease database on prevalence, incidence, Years Lived with Disability (YLDs), Years of Life Lost (YLLs), and Disability-Adjusted Life Years (DALYs), for brain disorders in Norway in 2023, with changes from 1990 to 2023.

**Findings:**

Brain disorders accounted for 26·64% of all DALYs, 30·77% of all YLDs and 22·31% of all YLLs in Norway in 2023, no uncertainty intervals (UIs) given. The highest burden in terms of DALYs was observed for Alzheimer's disease (AD) and dementias (3·82% of all DALYs, UI: 1·72–7·87), anxiety disorders (3·79%, UI: 2·77–4·97), stroke (3·43%, UI: 3·04–3·89), headache (2·5%, UI: 1·94–3·18), and depressive disorders (2·22%, UI: 1·71–2.90). Leading causes of YLDs were anxiety (7·35%, UI: 5·35–9·65), headache (4·91%, UI: 3·91–5·88), and depression (4·29%, UI: 3·47–5.42), while AD and dementias (5·47%, UI: 1·35–13·54), stroke (5·46%, UI: 4·75–5·95), and self-harm (3·51%, UI: 3·17–3·97) were important causes of YLLs. Age-standardized DALYs decreased over the 34 years for stroke, meningitis, and alcohol use disorders, whereas Parkinson's disease, anxiety, depressive, eating, and drug use disorder increased.

**Interpretation:**

Brain disorders are causing more than a quarter of all disease burden in Norway, which is of importance for prioritizing resources to health services and to research. Although there are concerning trends for some disorders, others have become considerably less burdensome since 1990, likely due to improvements in general health, prevention, and effective new treatments and rehabilitation.

**Funding:**

The Gates foundation and Norwegian Institute of Public Health.

**Research in context**

**Evidence before this study**

The study was based on the concept ‘Brain disorders’, which has primarily been used in connection with health economic analyses by the European Brain Council, and which includes the main neurological, neurosurgical, mental and addiction disorders. We searched PubMed for studies investigating the total burden of these disorders in terms of Years Lived with Disability (YLDs), Years of Life Lost (YLLs), and the sum of these, Disability-Adjusted Life Years (DALYs). These are measures of disease burden used by the Global Burden of Disease (GBD) Study. We wished to identify studies with data on brain disorders in a single country, a region, or globally, by searching PubMed from database inception through August 24th, 2026, using the search terms (“Brain disorders”) AND (“Disease burden”) AND (“GBD”), and then reading through all titles and in some cases also abstracts. Many studies had investigated disease burden of one or a few of these disorders, but only one had investigated the total burden of all brain disorders. This study, based on GBD in the year 2000, reported the disease burden of brain disorders in three regions of Europe. In the region roughly corresponding to Western Europe to which Norway belonged, brain disorders accounted for 37% of the total disease burden, more in terms of YLDs (54%) and less in terms of YLLs (18%). No study had investigated how the burden of all these disorders had changed over a time period.

**Added value of this study**

This is the first study of the total burden of brain disorder using GBD 2023 data and methodology for any single country. It shows that the burden is large in Norway (27% of all DALYs), but somewhat less than in the study based on GBD 2000 for Western Europe. Brain disorders are more important as cause of disability (31% of all YLDs) than of mortality (22% of YLLs). The study is also the first to report changes in the burden of brain disorders over time. From 1990 till 2023 there was a marked decline in prevalence and burden of stroke, meningitis, and alcohol use disorder when adjusting for population growth and aging, but a steep increase in disease burden for particularly Parkinson's disease, but also for anxiety disorders, depressive disorders, drug use disorders and eating disorders.

**Implications of all the available evidence**

Since 1990, there have been marked positive changes regarding burden of some brain disorders in Norway, probably caused by improvements in general health, as well as new targeted treatments, prevention, and rehabilitation measures. For other brain disorders, there is a worrying and mainly unexplained increased burden. With the continued aging of the population brain disorders will pose a large challenge to population health in the near future. Monitoring brain health through studies like the GBD study is necessary to evaluate the overall effects of preventive measures and new and costly treatments and other interventions, as well as of general changes in population health. It is also essential for tailoring capacity of health services, pinpointing emerging challenges, and directing research priorities.

## Introduction

The non-profit organization ‘The European Brain Council’ (EBC) was founded in 2002 with the aim to “unite the brain community and promote brain research with the goal of improving the lives of these who live with a brain condition, neurological and mental alike”.^1^ When EBC was founded, the concept of brain disorders was a new one.^2^ From the outset, it included all disorders, conditions and injuries that affected the brain and nervous system, i.e. conditions traditionally regarded as neurological, psychiatric, or neurosurgical. This delimitation of brain disorders accords well with those included under the term “brain health”,^3^ used in in the recent Brain Health Strategy of the Norwegian Ministry of Health and Care Services.^4^ This comprehensive strategy was developed because these disorders have several common denominators and constitute a substantial, but often underestimated, societal health burden. For the EBC, advocacy has been a main reason for grouping all brain disorders under the same heading. Both professional and patient organizations recognized that these disorders placed a significant burden on individuals and society, yet they received little attention and inadequate funding to research and treatment compared to diabetes, cancer^5^ and cardiovascular diseases.^2^ Another equally important reason for grouping them together was the potential for new insights, both basic and clinical, to benefit multiple disorders with distinctly different manifestations.^6^

Data from the Global Burden of Diseases, Injuries and Risk Factors Study (GBD) for the year 2000 (GBD 2000) has been an important premise for the foundation of the EBC. The GBD studies measures disease burden as health loss due to both mortality and disability by time units, i.e. number of Years of Life Lost (YLLs), Years Lived with Disability (YLDs), and the sum of the two, Disability-Adjusted Life Years (DALYs). In a study based on GBD 2000, brain disorders were found to account for as much as 37% of the total disease burden (DALYs) in Western Europe.^2^

Studies based on GBD with global and national data have previously been published for neurological disorders (GBD2021) and for mental disorders (GBD2019).^7^^,^^8^ Previously, the concept of brain disorders has only been used in a few health economic analyses, in Europe and globally.^9^^,^^10^ To our knowledge, no previous studies have investigated the collective burden, in terms of DALYs, YLDs, and YLLs, of brain disorders globally, or in a single country, with updated GBD data and methodology. Norway is a typical example of a Western European country with a well-developed public health and welfare system, a relatively homogeneous population, and substantial immigration during the last decades. Precise estimates of the burden of brain disorders would be of considerable interest. The Norwegian Directorate of Health published a Brain Health Strategy in 2017 in an attempt to better deal with the challenges of the brain disorders, using the same definition as outlined by EBC.^4^ Precise knowledge about the nationwide overall impact of all brain disorders including mental health and addiction is much needed for health strategies and planning. The aim of the present study was to utilize data from the most recent iteration of GBD (GBD 2023)^11^ to assess the prevalence, incidence and burden of brain disorders in Norway, both overall and for individual disorders, for all ages and both sexes. Additionally, the study examined time trends of this burden over the past 34 years (1990–2023).

## Methods

The Global Burden of Disease Study (GBD) is a comprehensive research project that quantifies health loss from diseases, injuries and risk factors across populations and over time. A substantive description of the methods behind the disease burden analyses is provided in the DALY capstone article and its appendices from GBD 2023.^11^ An overview of the methods relevant for the current study is provided below.

GBD cause categorization has been based on, but is not identical to, the WHO-based International Classification of Diseases (ICD),^12^ and to some degree on the Diagnostic and Statistical Manual of Mental Disorders.^13^ In GBD, there is a hierarchical classification of diagnoses (“causes”), with 4 levels representing increasing diagnostic granularity. At Level 1, there are three broad cause groups (1: Communicable, maternal, neonatal, and nutritional diseases, 2: Non-communicable diseases, and 3: Injuries). At level 2 there are more specified disease categories (e.g. neurological disorders, mental disorders), while specific diseases are detailed on level 3 (e.g. headache disorders and depressive disorders), and level 4 (e.g. migraine and major depressive disorder). GBD has no overall category for brain disorders. Most of these disorders can be found under the heading of neurological disorders and mental disorders. The neurological disorders include Alzheimer's disease (AD) and dementia, Parkinson's disease (PD), idiopathic epilepsy, multiple sclerosis, motor neuron disease (MND), headache (with subcategories migraine and tension-type (TTH) headache), and other neurological disorders (OND, consisting of primary muscle disorders, myasthenia gravis, Huntington's disease). The mental disorders include schizophrenia, depressive disorder (with subcategories major depression and dysthymia), bipolar disorder, anxiety disorders, eating disorders (with subcategories anorexia nervosa and bulimia nervosa), attention deficit hyperactivity disorder (ADHD), autism spectrum disorder, conduct disorder, idiopathic developmental intellectual disability (IDID), and other mental disorders (OMD, mostly personality disorders). However, some important disorders were classified based on their underlying etiology, e.g. stroke under cardiovascular diseases, brain and spinal cord injury under injuries, meningitis and encephalitis under infectious diseases, and brain and CNS cancers under neoplasms. Several conditions related to mental disorders are classified in GBD under other headings, such as alcohol and drug use disorders (under substance use disorder), and self-harm (under injuries). In the latest disease classification (ICD-11), cerebrovascular disorders (stroke) are classified as a nervous system disease, along with the traditional neurological disorders.^12^

For the present study, all data on prevalences, incidences, YLLs, YLDs, and DALYs (Table 1, Table 2, Table 3, Table 4) for the different disorders were extracted from the GBD website GBD Results Tool, on the website of the Institute of Health Metrics and Evaluation (IHME),^14^ except the summary figures for DALYs, YLDs, and YLLs of all brain disorders (see below). The methods of the GBD have been described recently,^15^^,^^16^ and an overview has been published in Norwegian.^17^ GBD collects data on incidence, prevalence, disease durations, severity, and excess mortality through systematic literature searches, proactive data seeking and via the GBD collaborator network.^16^ For GBD 2023, more than 100,000 data sources globally were included to generate DALY estimates. Important data sources for Norway include vital registrations (the Norwegian Cause of Death registry) and patient registry data, as well as data from epidemiological surveys and various health statistics. Underlying data sources for Norway in GBD 2023 are found on the Global Health Data Exchange (GHDx) website,^18^ and listed in Appendix 1. A Bayesian meta-regression statistics tool (DisMod-AT, Disease rates Modeled as functions of Age and Time)^11^ adjusts for known biases with correction factors, e.g. changes in diagnostic practices, and models internally consistent estimates of prevalence, incidence, remission and mortality for each sex, age-group, year and location. A hierarchical cascade model for estimates is used, where all global data inform a global estimate, which is then used as a prior for a super-region estimate. The priors then follow down in the hierarchy with increasingly more geographical detail (global, super-regional, regional, national, and subnational) with adjustments from local data and covariates. This cascade model ensures that estimates are generated also for locations where local data are missing.

**Table 1:** Prevalence (percent of population) and incidence (rate per 100,000) of brain disorders in Norway 2023, with percent change in age-standardized rates from 1900 to 2023.

Disorder	Prevalence				Incidence			
	% of population		% Change from 1990^a^		Rate^b^ in 2023		% Change from 1990^c^	
	95% UIs		95%UI		95% UI		95%UI	
**Neurological**	51·52	47·82–54·88	1	0–3	13,718	12,174–15,314	1	−2 to 4
AD and dementia	1·66	1·37–1·91	−5	−9 to −3	280	234–320	−4	−8 to −2
PD	0·24	0·20–0·30	271	238–306	27	23–32	115	95–135
Idiopathic epilepsy	0·45	0·28–0·61	9	−26 to 49	54	33–77	8	−26 to 49
Multiple sclerosis	0·20	0·17–0·24	44	37–52	6	5–7	33	26–41
MND	0·02	0·02–0·02	18	14–22	4	4–4	9	7–11
Headache	50·01	46·35–53·36	1	−1 to 3	13,346	11,792–14,920	*1*	−2 to 4
*Migraine*	*18·04*	*15·65–20·47*	*5*	*2*–*8*	*1149*	*1027–1295*	*5*	*2–7*
*TTH*	*40·01*	*36·06–44·43*	*0*	−3 to 3	*12,196*	*10,734–13,752*	*0*	−3 to 4
OND	0·00	0·00–0·00	−29	−48 to 5	1	1–1	−29	−51 to 13
Cerebrovascular (Stroke)^d^	1·67	1·47–1·84	−28	−31 to −25	140	124–160	−52	−55 to −48
*Ischemic*	*1·33*	*1·13–1·50*	−30	−33 to −26	105	90–124	−56	−59 to −51
*Hemorrhage*	*0·18*	*0·16–0·21*	−16	−21 to −10	23	19–27	−36	−40 to −30
*SAH*	*0·17*	*0·15–0·20*	−30	−33 to −28	12	10–14	−36	−39 to −32
**Mental**	17·69	15·85–19·58	29	13–55	5038	4469–5638	23	7–45
Schizophrenia	0·35	0·30–0·40	0	−1 to 2	18	15–21	0	−2 to 2
Depressive	3·73	3·31–4·27	26	7–52	3489	3028–3978	19	2–43
*Major depression*	*2·68*	*2·28–3·17*	*41*	*11*–*83*	*3333*	*2875–3808*	*20*	*2–46*
*Dysthymia*	*1·08*	*0·92–1·25*	*0*	0–0	*155*	*127–190*	*0*	*0*–*0*
Bipolar disorder	0·70	0·60–0·81	0	−2 to 2	38	32–44	0	−2 to 1
Anxiety disorder	9·31	7·28–11·39	57	19–123	1052	791–1398	49	8–138
Eating disorders	0·56	0·45–0·74	18	15–21	268	181–398	14	10–18
*Anorexia nervosa*	*0·12*	*0·09–0·15*	*18*	*13–22*	*27*	*20–37*	*13*	*8–19*
*Bulimia nervosa*	*0·45*	*0·33–0·62*	*189*	*14–23*	*241*	*154–375*	*14*	*10–19*
ADHD	0·62	0·44–0·87	0	0–0	26	17–39	0	0–0
Autism spectrum	0·74	0·43–1·20	18	−33 to 155	10	3–25	38	−61 to 319
Conduct	0·44	0·32–0·56	0	0–0	137	102–174	0	0–0
IDID	0·42	0·16–0·85	0	−31 to 58	54	33–77	8	−26 to 49
OMD	2·03	1·63–2·51	0	0–0	0	0–0	0	0–0
**Substance use**	2·64	2·41–2·87	−25	−28 to −22	1065	918–1206	−31	−34 to −27
Alcohol use	1·59	1·39–1·81	−43	−45 to −41	740	618–872	−42	−45 to −40
Drug use	1·07	0·96–1·18	23	12–31	325	277–392	19	12–26
OTHER^e^								
Self-harm	0·29	0·26–0·34	2	−1 to 4	151	129–175	2	−2 to 9
CNS cancer	0·13	0·10–0·17	58	0–168	20	16–25	35	−1 to 95
Meningitis	0·04	0·03–0·05	−62	−64 to −59	11	9–14	−56	−59 to −53
Encephalitis	0·01	0·01–0·02	−19	−22 to −16	7	6–8	−4	−6 to −2
Traumatic brain injury	0·89	0·83–0·94	−1	−4 to −2	485	355–651	0	−9 to −8
*Minor*	0·53	0·48–0·58	−3	−8 to −1	353	244–510	−1	−12 to −8
*Moderate/severe*	0·36	0·33–0·40	3	−2 to 8	132	93–183	6	−5 to 8
Spinal Cord Injury	0·69	0·61–0·79	4	−3 to 11	20	13–31	2	−13 to 15
*Neck level*	0·38	0·33–0·43	7	0–13	12	7–21	2	−19 to 17
*Below neck*	0·31	0·27–0·35	0	−7 to 7	8	4–15	1	−20 to 18

Data from GBD 2023. The categories in **bold text** are level 2 diagnoses, in plain text are level 3, and in *italics* are level 4.

CNS: Central nervous system, AD: Alzheimer's disease, PD: Parkinson's disease, MND: Motor neuron disease, TTH: Tension-type headache, OND: Other neurological disorders (primary muscle disorders, myasthenia gravis, and Huntington's disease), SAH: Subarachnoid hemorrhage, ADHD: Attention deficit/Hyperactivity disorder, IDID: Idiopathic developmental intellectual disability, OMD: Other mental disorders (mostly personality disorders), UI: Uncertainty intervals.

a (Prevalence 2023-prevalence 1990)/prevalence 1990∗100.

b Per 100,000 population.

c (Incidence rate 2023-incidence rate1990)/incidence rate 1990∗100. Prevalence and incidence rates in^a^ and^c^ are age standardized.

d A level 3 diagnosis under cardiovascular diseases in GBD.

e These are disorders categorized under various level 2 diagnoses by GBD (injuries, neoplasms, and infectious diseases).

**Table 2:** Disability-adjusted life years (DALYs) in percent of total DALYs and rate per 100,000 for different brain disorders in Norway in 2023, with changes in age-standardized rates from 1990.

Disorder	% of total DALYs		DALYs rate^a^		% Rate change from 1990^b^	
	95% UIs		95%UI		95%UI	
All brain disorders	26·64		7289			
**Neurological**	8·96	6·85–12·93	2448	1736–3579	6	1–11
AD and dementia	3·82	1·72–7·87	1042	476–2121	−4	−9 to 0
PD	0·74	0·64–0·84	200	175–219	43	32–54
Idiopathic epilepsy	0·53	0·38–0·77	145	102–225	−13	−33 to 12
Multiple sclerosis	0·35	0·30–0·41	96	78–115	14	−1 to 35
MND	0·31	0·26–0·36	85	72–98	17	−4 to 42
Headache	2·53	1·94–3·18	694	483–940	4	2–7
*Migraine*	*2·23*	*1·64–2·83*	*611*	*410–859*	*5*	*2–8*
*TTH**^2^*	*0·30*	*0·22–0·40*	*83*	*57–117*	*3*	−1 to 6
OND	0·68	0·60–0·78	187	169–206	64	47–81
Cerebrovascular (Stroke)^c^	3·43	3·04–3·89	943	827–1026	−65	−69 to −63
*Ischemic*	*2·08*	*1·81–2·36*	*569*	*482–643*	−71	−74 to −68
*Hemorrhage*	*0·92*	*0·77–1·07*	*250*	*221–279*	−54	−61 to −47
*SAH*	*0·46*	*0·40–0·52*	*124*	*110–139*	−56	−62 to −49
**Mental**	9·05	7·37–10·81	2482	1830–3233	31	14–54
Schizophrenia	0·76	0·58–0·95	208	156–263	1	−3 to 3
Depressive	2·22	1·71–2·90	609	427–843	32	9–65
*Major depression*	*1·86*	*1·42–2·44*	*510*	*353–722*	*41*	*11–83*
*Dysthymia*	*0·36*	*0·27–0·46*	*98*	*67–141*	−*1*	−2 to 1
Bipolar disorder	0·52	0·37–0·71	142	92–197	0	−3 to 3
Anxiety disorder	3·79	2·77–4·97	1040	690–1476	58	19–122
Eating disorders	0·41	0·28–0·58	113	66–172	17	14–21
*Anorexia nervosa*	*0·09*	*0·06–0·12*	*24*	*14–36*	*15*	*9–22*
*Bulimia nervosa*	*0·32*	*0·20–0·49*	*89*	*50–136*	*18*	*13–23*
ADHD	0·09	0·06–0·13	25	15–37	0	−2 to 2
Autism spectrum	0·48	0·26–0·79	132	69–218	19	−34 to 157
Conduct	0·19	0·12–0·28	51	29–81	0	−3 to 3
IDID	0·07	0·03–0·16	21	8–42	−13	−33 to 12
OMD	0·52	0·37–0·69	141	94–202	0	−2
**Substance use**	2·60	2·28–2·91	709	586–827	−8	−15 to 0
Alcohol use	0·95	0·82–1·07	259	211–322	−50	−55 to −46
Drug use	1·65	1·39–1·91	450	374–537	58	43–79
OTHER^d^						
Self-harm	1·75	1·46–2·04	475	428–535	−37	−46 to −25
CNS cancer	0·79	0·64–0·94	215	189–242	−11	−25 to 6
Meningitis	0·03	0·03–0·04	9	7–10	−89	−91 to −88
Encephalitis	0·03	0·02–0·03	7	6–9	9	−14 to 33

Data from GBD 2023. The categories in **bold text** are level 2 diagnoses, in plain text are level 3, and in *italics* are level 4.

CNS: Central nervous system, AD: Alzheimer's disease, PD: Parkinson's disease, MND: Motor neuron disease, TTH: Tension-type headache, OND: Other neurological disorders (primary muscle disorders, myasthenia gravis, Huntington's disease), SAH: Subarachnoid hemorrhage, ADHD: Attention deficit/Hyperactivity disorder, IDID: Idiopathic developmental intellectual disability, OMD: Other mental disorders (mostly personality disorders), UI: Uncertainty intervals.

a DALYs per 100,000 population.

b (Rate 2023 -rate 1990)/rate 1990∗100. Rate changes were age-adjusted to remove differences caused by change in population age composition from 1990 to 2023.

c A level 3 diagnosis under cardiovascular diseases in GBD.

d These are disorders categorized under various level 2 diagnoses by GBD (injuries, neoplasms, and infectious diseases).

**Table 3:** Years lived with disability (YLDs) in percent of total YLDs and rate per 100,000 for different brain disorders in Norway in 2023, with changes in age-standardized rates from 1990.

Disorder	% of total YLDs		YLDs rate^a^		% Rate change from 1990^b^	
	95% UIs		95%UI		95%UI	
All brain disorders	30·77		4348			
**Neurological disorders**	8·62	6·89–10·35	1217	881–1604	1	−5 to 8
AD and dementia	2·27	1·69–2·91	320	220–414	−6	−10 to −4
PD	0·23	0·17–0·31	32	23–45	263	228–300
Idiopathic epilepsy	0·55	0·26–1·04	78	36–158	−10	−47 to 44
Multiple sclerosis	0·34	0·26–0·43	48	35–64	43	32–54
MND	0·03	0·02–0·03	4	3–5	17	14–21
Headache	4·91	3·91–5·88	694	483–940	4	2–7
*Migraine*	*4·33*	*3·28–5·33*	*611*	*410–855*	*5*	2–8
*TTH*^b^	*0·58*	*0·44–0·74*	*83*	*57–117*	3	−1 to 6
OND	0·29	0·22–0·38	41	28–57	178	122–242
Cerebrovascular (Stroke)^c^	1·58	1·27–1·90	223	158–290	−28	−31 to −24
*Ischemic*	*1·25*	*0·99–1·51*	*176*	*125–231*	−29	−33 to −25
*Hemorrhage*	*0·18*	*0·14–0·22*	*25*	*19–31*	−15	−21 to −8
*SAH*	*0·16*	*0·12–0·20*	*23*	*16–30*	−30	−34 to −26
**Mental disorders**	17·56	14·72–20·90	2482	1829–3233	31	14–54
Schizophrenia	1·49	1·10–1·97	208	156–263	0	−3 to 3
Depressive disorders	4·29	3·47–5·42	609	427–843	32	9–65
*Major depression*	*3·60*	*2·79–4·70*	*510*	*353–722*	*41*	*11–83*
*Dysthymia*	*0·69*	*0·55–0·85*	*98*	*67–141*	*0*	−2 to 1
Bipolar disorder	1·00	0·69–1·42	142	92–197	0	−3 to 3
Anxiety disorder	7·35	5·35–9·65	1040	690–1476	58	19–122
Eating disorders	0·79	0·57–1·07	113	65–171	18	14–22
*Anorexia nervosa*	*0·17*	*0·12–0·23*	*24*	*14–35*	*17*	*11–24*
*Bulimia nervosa*	*0·63*	*0·43–0·91*	*89*	*50–136*	*18*	*13*–*23*
ADHD	0·18	0·12–0·25	25	15–37	0	−2 to 2
Autism spectrum disorder	0·95	0·48–1·58	132	69–218	19	−34 to 157
Conduct disorder	0·36	0·23–0·52	51	29–81	0	−3 to 3
IDID	0·15	0·06–0·29	21	8–42	−10	−47 to 44
OMD	1·00	0·72–1·35	141	94–202	0	−2 to 2
**Substance use disorder**	2·83	2·29–3·32	399	286–504	−7	−13 to 0
Alcohol use disorder	1·05	0·86–1·28	149	103–209	−43	−45 to 41
Drug use disorder	1·77	1·34–2·21	250	178–317	40	32–49
OTHER^d^						
Self-harm	0·08	0·07–0·10	12	8–16	4	1–7
CNS cancer	0·07	0·06–0·1	10	7–15	42	−4 to 120
Meningitis	0·02	0·02–0·03	3	2–4	−59	−62 to −56
Encephalitis	0·01	0·01–0·01	1	1–2	−16	−18 to −13
Traumatic brain injury	0·78	0·66–0·90	110	77–149	−2	−5 to 2
*Minor*	0·42	0·35–0·49	59	40–81	−4	−9 to 1
*Moderate/severe*	0·36	0·30–0·43	51	36–71	2	−3 to 7
Spinal Cord Injury	1·28	0·98–1·56	180	123–236	2	−4 to 9
*Neck level*	0·94	0·69–1·17	132	91–169	5	−2 to 11
*Below neck level*	0·34	0·27–0·41	48	31–68	−5	−12 to 2

Data from GBD 2023. The categories in **bold text** are level 2 diagnoses, in plain text are level 3, and in *italics* are level 4.

CNS: Central nervous system, AD: Alzheimer's disease, PD: Parkinson's disease, MND: Motor neuron disease, TTH: Tension-type headache, OND: Other neurological disorders (primary muscle disorders, myasthenia gravis, Huntington's disease), SAH: Subarachnoid hemorrhage, ADHD: Attention deficit/Hyperactivity disorder, IDID: Idiopathic developmental intellectual disability, OMD: Other mental disorders (mostly personality disorders). UI: Uncertainty intervals.

a YLDs per 100,000 population.

b (Rate 2023-rate 1990)/rate 1990∗100. Rate changes were age-adjusted to remove differences caused by change in population age composition from 1990 to 2023.

c A level 3 diagnosis under cardiovascular diseases in GBD.

d These are disorders categorized under various level 2 diagnoses by GBD (injuries, neoplasms, and infectious diseases).

**Table 4:** Years of life lost (YLLs) in percent of total YLLs and rate per 100,000 for different brain disorders in Norway in 2023, with changes in age-standardized rates from 1990.

Disorder	% of total YLLs		YLLs rate^a^		% Rate change from 1990^b^	
	95% UIs		95%UI		95%UI	
All brain disorders	22·31		2941			
**Neurologic disorders**	9·34	5·42–17·17	1231	718–2280	5	−3 to 16
AD and dementia	5·47	1·35–13·54	721	178–1798	−3	−9 to 4
PD	1·28	1·13–1·38	168	148–182	27	18–35
Idiopathic epilepsy	0·51	0·44–0·59	67	58–77	−17	−34 to 2
Multiple sclerosis	0·36	0·29–0·44	48	39–59	−7	−29 to 24
MND	0·62	0·53–0·72	81	70–95	17	−5 to 43
OND	1·11	1·01–1·20	146	133–159	47	31–64
Cerebrovascular (Stroke)^c^	5·46	4·75–5·95	720	630–788	−71	−74 to −68
*Ischemic*	*2·98*	*2·48–3·34*	*393*	*326–438*	−78	−80 to −75
*Hemorrhage*	*1·71*	*1·51–1·93*	*225*	*199–255*	−57	−64 to −49
*SAH*	*0·77*	*0·68–0·87*	*102*	*90–114*	−59	−66 to −51
**Mental disorders**	0·00	0·00–0·01	0·58	0.06–1.02	−35	−66 to 58
Eating disorders	0·00	0·00–0·01	0·58	0.06–1.06	−35	−66 to 58
*Anorexia nervosa*	0·00	0·00–0·01	0·58	0.06–1.06	−35	−66 to 58
**Substance use disorder**	2·36	2·06–2·67	310	270–356	−9	−22 to 10
Alcohol use disorder	0·83	0·72–0·97	110	95–128	−60	−67 to −51
Drug use disorder	1·52	1·24–1·83	200	163–244	97	52–149
OTHER^d^						
Self-harm	3·51	3·17–3·97	463	416–524	−37	−46 to −26
CNS cancer	1·56	1·37–1·76	205	181–231	−12	−26 to 4
Meningitis	0·04	0·04–0·05	5	5–6	−92	−93 to −91
Encephalitis	0·04	0·04–0·05	6	5–7	20	−13 to 62

Data from GBD 2023. The categories in **bold text** are level 2 diagnoses, in plain text are level 3, and in *italics* are level 4. Disorders with no YLLs have been omitted.

CNS: Central nervous system, AD: Alzheimer's disease, PD: Parkinson's disease, MND: Motor neuron disease, OND: Other neurological disorders (primary muscle disorders, myasthenia gravis, Huntington's disease), SAH: Subarachnoid hemorrhage. UI: Uncertainty intervals.

a YLLs per 100,000 population.

b (Rate 2023 -rate 1990)/rate 1990∗100. Rate changes were age-adjusted to remove differences caused by change in population age composition from 1990 to 2023.

c A level 3 diagnosis under cardiovascular diseases in GBD.

d These are disorders categorized under various level 2 diagnoses by GBD (injuries, neoplasms, and infectious diseases).

Estimates of YLLs for causes of deaths (See Table 4) are calculated by subtracting the age of death from the remaining age-specific life expectancy at time of death. The age-specific life-expectancy is retrieved from a life table based on the longest observed life expectancy globally in populations of more than 5 million. For many conditions, like most of the mental disorders and also the headaches, mortality is set at 0 in the statistical modeling. Mortality related to such disorders may partly be captured by the category ‘self-harm’.^19^

YLDs are generated as the product of disease prevalence multiplied with a corresponding disability weight (DW) ranging from 0 (no health loss) to 1 (health loss equal to being dead). The DWs represent an attempt to quantify the health loss associated with different diseases, taking severity into account, and are generated from community surveys among tens of thousands of ordinary citizens in several countries and of widely different cultures, based on short lay descriptions of disease states given in different languages.^20^^,^^21^ YLD estimates were corrected for independent comorbidities, and for individuals with several comorbid conditions, a combined DW was calculated in a multiplicative rather than an additive way, to avoid the total DW exceeding 1. When calculating the total YLDs of a disorder in the population, only the fraction of the YLD attributable to the disorder in each individual was taken into account, to avoid overestimations at the population level.^7^^,^^22^ DALYs are calculated as the sum of YLLs and YLDs.

Through comorbidity corrections and the multiplicative way DWs are calculated in individuals with several disorders, the GBD methodology ensures that the sum of all disorders at each diagnostic level adds up to 100%. Therefore, adding the burden of each disorder to obtain a measure of the total burden of brain disorders will not lead to overestimation. The same is true for the rates (DALYs/YLDs/YLLS per 100,000 population). For prevalence figures the sum of prevalences of the individual disorders are allowed to exceed the total patient population, even with comorbidity corrections.

Details of the modeling for all disorders including those treated in this paper, can be found in Appendix 1 to reference.^11^ Estimates produced by the GBD study are compliant with the Guidelines for Accurate and Transparent Health Estimates Reporting (GATHER, see Appendix 2).

For the present study, aiming to show the importance of brain disorders relative to all other disorders in Norway, it was most relevant to present DALYs, YLDs, and YLLs for each disorder as percent of all DALYs, YLDs, and YLLs. For comparative purposes we have only given rates per 100,000 and not absolute numbers. The latter can be calculated from the rates for the total Norwegian population of 5,470,982 in Norway in the middle of 2023.^14^ Absolute numbers are useful for assessing total health burden in Norway and thereby for planning of services.

All data were extracted independently by two of the authors (LJS and AKSK) during October and November 2025. Discrepancies were checked and corrected during January 2026. The data were extracted on the national level, encompassing both sexes and all age groups, showing the disease burden with the age and sex distribution as it was in 2023. When analyzing changes from 1990 to 2023, the data were age-standardized to account for differences in the population's age composition between these two time points. All data were given with 95% uncertainty intervals (UIs).

Since “Brain disorders” is not a disease category in GBD, the burden data for this category could not be extracted directly from the GBD results tool but were calculated by the authors as the sum of the underlying disorders. Hence, the burden figures for Brain Disorders are without uncertainty intervals.

For injuries, GBD mostly presents data according to their cause (e.g. falls, vehicle accidents, violence etc., considered most relevant for injury prevention) and not to their anatomical location, except regarding prevalence, incidence and YLD. Hence, for spinal cord injury and head trauma, only these three measures of burden were available. Therefore, when summarizing DALYs, YLDs, and YLLs, these traumatic disorders were not included.

### Role of the funding source

The funding sources had no role in the study design, data collection, data analysis, interpretation, or writing of the report.

## Results

Estimated prevalences and incidences for each brain disorder in Norway in 2023, and the percent (%) change (age-standardized) from 1990 are given in Table 1. Neurological disorders affected 52% of the population, with headache disorders, including both migraine and tension-type headache (TTH), as the most common level 3 cause (prevalence 50·01%). Alzheimer's disease (AD) and dementia affected 1·66% and stroke 1·67%. Mental disorders affected 17·69%, the most prevalent level 3 causes being anxiety (9·31%) and depressive disorders (3·73%). Substance use disorders affected 2·64%, with alcohol use disorder being more prevalent than drug use disorder (1·59% versus 1·07%).

**Fig. 1:**
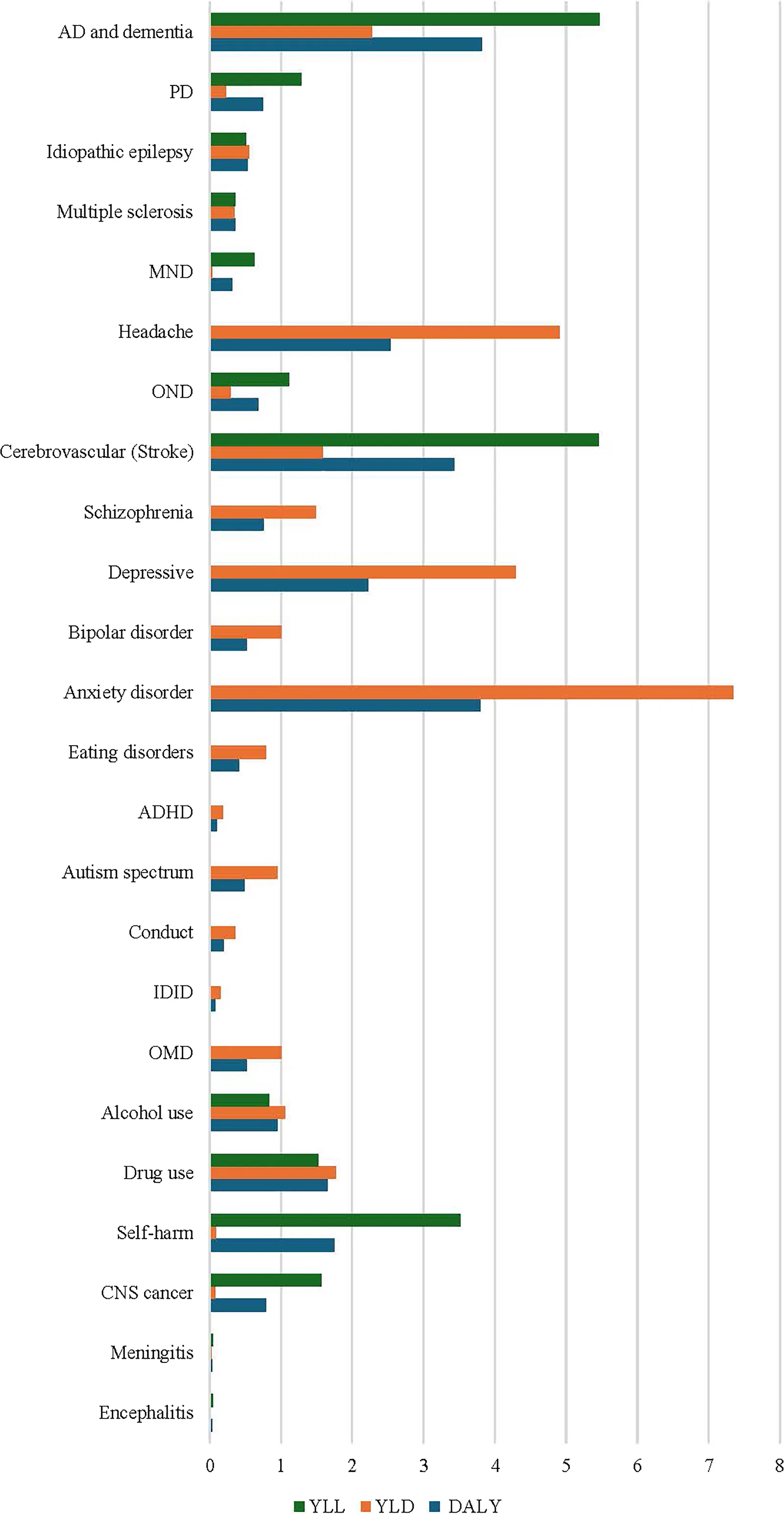
DALYs, YLDs, and YLLs of all brain disorders in Norway in 2023, as % of DALYs, YLDs, and YLLs of all disorders.

All brain disorders combined accounted for 26·64% of all DALYs (Table 2), 30·77% of all YLDs (Table 3), and 22.31% of all YLLs (Table 4). At GBD level 2, mental disorders were the largest contributor to DALYs (9·05%), followed by neurological disorders (8·96%), and substance use disorders (2·36%). Among the more detailed level 3 causes, the five largest causes of DALYs among the brain disorders were Alzheimer's disease and dementia (3·82%), stroke (3·43%), anxiety disorders (3·79%), headache disorders (2·53%), and depressive disorders (2·22%). The relative importance of the different brain disorders in terms of DALYs, YLDs, and YLLs is shown in Fig. 1.

The brain disorders differed in the impact they had on disability and mortality (YLDs and YLLs). On GBD level 2, mental disorders and neurological disorders had the largest impact on YLDs (17·56% and 8·62% of all YLDs, respectively), with anxiety disorders, headache disorders and depressive disorders as major specific level 3 causes (Table 3). In terms of mortality, neurological disorders were the most important group (9·34% of all YLLs), primarily due to AD and dementias. Stroke, self-harm, and CNS cancer were also important level 3 causes of YLLs among the brain disorders (Table 4).

Figures S1, S1b, S2–S5 (Appendix 3) show brain disorders (only GBD level 3) sorted according to prevalence, incidence, DALYs, YLDs, and YLLs. In Figures S6–S10 the same disorders are sorted according to the age-standardized change in these parameters from 1990 till 2023. Looking at changes in prevalence and incidence and considering only disorders with UIs not including 0 (Table 1 and Figure S1 and S2), the most notable change over time is for Parkinson's disease (PD), for which prevalence increased by 271% and incidence by 115%. Other disorders with a marked increase were anxiety disorder (57% and 49%), multiple sclerosis (44% and 33%), depressive disorder (26% and 19%), drug use disorder (23% and 19%), eating disorders (18% and 14%), and motor neuron disease (MND) (18% and 9%). Some diseases had a decrease, such as meningitis (−62% and −56%), alcohol use disorder (−43% and −42%), stroke (−28% and −52%), encephalitis (−19% and −4%), and AD and dementia (−5% and −4%).

Changes of prevalence and incidence over time were usually reflected in changes in disease burden (Table 2, Table 3, Table 4 and Figures S6–S10) with significant increases in DALY, YLD, and YLL rates of anxiety (58%, 58%, no mortality), drug use disorder (58%, 40%, and 97%), PD (43%, 263%, and 27%), and depressive disorders (32%, 32%, no mortality). Similarly, there was a decrease in meningitis (−89%, −59, and −92%), stroke (−65%, −28%, and −71%), and alcohol use disorder (−50, −43, and −60).

For eating disorders, an increase in prevalence and incidence was reflected in increasing DALYS and YLDs (17%, 18%), but a decrease in YLLs (−35%). Also, for some other disorders, there was a discrepancy between the prevalence/incidence figures and burden figures. For self-harm, there were stable figures for prevalence, incidence and YLDs rates, but significant decreases in DALYs and YLLs (−37, −37). The rare and heterogenous disorders included in the category Other Neurological Disorders (OND: primary muscle disorders, myasthenia gravis, and Huntington's disease) had collectively no significant prevalence and incidence changes, but a marked increase in DALYs, YLDs, and YLLs (64%, 179%, and 47%).

For Idiopathic developmental intellectual disability (IDID) and autism spectrum disorder, the UIs were very large for both prevalence and incidence, leading to large UIs also for all the burden measures.

## Discussion

The present paper shows that in 2023, brain disorders constituted 27% of the total disease burden (DALYs) in Norway, and more than 4347 healthy life years (YLDs) were lost due to the non-fatal consequences of brain disorders which comes in addition to the 2940 YLLs caused by premature deaths from these disorders. Because brain disorders were more responsible for conditions we suffer from (30·77% of YLDs) than for those we die from (22·31% of YLLs), brain disorders are particularly important from a socioeconomic perspective.

To our knowledge no other studies give data on the collective burden of brain disorders from other countries or regions that are directly comparable to the data given here. The DALY figures for brain disorders in Norway are considerably lower than those in the study based on GBD 2000 for Western Europe (37%), while YLD figures are much higher (54%) and YLL figures lower (18%).^2^ However, speculating on the cause of these discrepancies is futile since the GBD methods have changed profoundly and the number of conditions included have increased dramatically. It would be of interest to compare our results with recent GBD data from other countries, both in Europe and elsewhere. This could contextualize our findings and give rise to hypotheses about important determinants of brain health and detection of modifiable risk factors.

Looking at the time-trends (Tables and Figures S6–S10), it is encouraging that one of the most burdensome disorders overall, stroke, had a marked decline (−65%) in age-standardized DALY rates (Figure S8). This can be explained by reduced incidence (−52%) and prevalence (−28%) as well as a markedly reduced mortality (YLLs rate, −71%), and also a reduction in disability (YLDs rate, −28%). This reduction was likely due to a decrease in cardiovascular diseases overall, caused by life-style changes (such as smoking cessation, less alcohol use, and a healthier diet), medications (such as blood pressure and cholesterol-lowering drugs, anti-platelet agents and anticoagulants), improved treatment of diabetes,^23^ better medical and surgical management of acute stroke,^24^ as well as more intensive and targeted rehabilitation for stroke patients.^25^

Interestingly, there were probably modest reductions in age-standardized AD and dementia disease burden (DALYs −4%, YLDs −6%, and YLLs −3%), although UIs for DALYs and YLLs included 0. This reduction reflects reduced prevalence and incidence (−5% and −4%). A similar trend has been noted in many parts of the world.^26^ The reduction may be due to the general improvement in cerebrovascular health,^27^ as vascular dementia and AD combined with vascular dementia (“Mixed dementia”), is common. Also, the marked reduction in alcohol use disorder (−43% in prevalence) probably plays a role.

No similar reduction was observed for other neurodegenerative disorders such as Parkinson's disease (PD) and motor neuron disease (MND), for which there have been no breakthroughs in prevention or treatment during the observation period. The age-standardized prevalence and incidence of PD increased dramatically (271% and 115%), and also MND saw an increase in prevalence and incidence (18% and 9%). As to the disease burden, this had increased markedly for PD (DALY 43%, YLD 263%, and YLL 27%) and somewhat less for MND (DALYs, YLDs, and YLLs 17%). The increase in the prevalence and burden of PD is concerning, and has been documented previously.^28^ Since mortality has risen far less than prevalence, the increase in prevalence may partly be attributed to improved survival rates due to better treatment and follow-up. However, this does not fully explain the increase, as there has also been a significant rise in age-standardized incidence of PD. Improved diagnosis, i.e. detecting more mild cases and/or cases earlier in the disease process may play a role. Other contributing factors might include unknown environmental toxins and smoking cessation in industrialized countries.^29^^,^^30^ Identifying the precise causes of the rise in PD incidence and prevalence should be a high priority, as they may be preventable.

For idiopathic epilepsy the observed changes were uncertain as the UIs included 0. The listed prevalence and incidence figures do not truly reflect the total burden of epilepsy because globally, idiopathic epilepsy accounts for only 47% of all cases with epilepsy, the remainder being secondary epilepsies, the burden of which is captured by the underlying disorder such as stroke, brain tumour and encephalitis.^31^ Treating the secondary epilepsies is more difficult and resource-demanding because of the concomitant, underlying disorders.

For multiple sclerosis (MS), the overall burden (DALYs) was relatively stable during the observation period. The increased prevalence and incidence (44% and 33%) could at least partly be accounted for by more sensitive diagnostic methods, capturing also mild and early cases,^32^ but it may also be caused by a decrease in mortality (YLL −7%, although UI includes 0), possibly caused by introduction of effective immune-modulating therapies during the time period.

For CNS cancer there was an increased incidence and prevalence (35% and 58%), in combination with increased YLDs (42%) but decreased DALYs and YLLs (−11, −12). These figures are somewhat uncertain as all UIs include 0. The trends can, at least partly, be accounted for by more sensitive diagnostic methods and improved treatment, leading to longer survival. Similar trends for brain cancer (higher incidence, lower mortality) have been demonstrated in other high-income countries.^33^

For both MS and CNS cancers, it would be valuable to examine how changes in diagnosis and treatment have contributed to the observed changes in prevalence, mortality and morbidity.

Headache disorders, particularly migraine, showed stable prevalence and incidence over time. This is in line with a large study repeated three times over more than 20 years in Norway.^34^ Despite the introduction of new acute and prophylactic treatments during this period, no difference in overall morbidity (YLDs) was observed. It would be valuable to explore whether this lack of impact is due to a modest efficacy of the drugs in real-life treatment, or results from limited access to these medications for the majority of individuals suffering from migraine.^35^

‘Other neurological disorders’ (OND, including primary muscle disorders, myasthenia gravis, Huntington's disease) were relatively stable regarding prevalence and incidence, but had a large increase in DALYs (64%), YLDs (178%), and YLLs (47%). To discern the drivers behind these changes, one should examine details about trends in diagnosis, treatments, morbidity, and mortality over these years for each of the disorders.

Among the mental disorders, there was an overall increase from 1990 to 2023 (prevalence 29% and incidence 23%), mostly driven by anxiety (53% and 49%), depressive (26% and 19%), and eating disorders (17% and 14%). This translated into similar increases in DALYs (mental 31%, anxiety 58%, depressive 32%, and eating disorders 17%) and YLDs (mental 31%, anxiety 58%, depressive 32%, and eating 18%). The same trends have been reported from UK.^36^ Part of this increase may be caused by changes in diagnostic practices and increased awareness over the period. None of the mental disorders showed an improvement over the time period, and the marked increase in mental health problems among young girls may be a contributing factor.^37^ Self-harm, a condition related with several of the mental disorders, showed stable prevalence, incidence and morbidity (YLDs) figures, but a decrease in mortality (YLLs down 37%). Stable rates of suicide attempts but fewer deaths due to suicides is a trend also seen globally.^15^ The reduction is most marked among men in Norway.^38^ This trend towards less self-harm deaths may reflect a shift toward more open discussions about suicide and severe mental disease, along with greater awareness of suicide risks among the public and healthcare professionals.

Regarding substance use disorders, there were opposite trends for alcohol and drug use. The prevalence and incidence of the former have been markedly reduced (−43% and −42%), whereas the latter increased (23% and 19%). These trends were reflected in similar changes in DALYs (−50%, 58%), YLDs (−43%, 40%), and YLLs (−60%, 97%). The alarming increasing mortality from drug use, probably in part reflects an increased use of potent opioids and multiple substance abuse.^39^

A success story is the decreased prevalence, incidence and mortality of CNS infections, particularly meningitis (−62%, incidence −56%, YLL -92%), but also encephalitis (− 19% and −4%), probably reflecting improved treatment (antibiotics, antiviral therapy) and prevention (vaccination). For encephalitis, prevalence and incidence have also decreased (−19% and −4%), but there is no significant drop in mortality or morbidity.

For some disorders (autism spectrum disorder, idiopathic developmental intellectual disability (IDID), and CNS cancer) there seem to be marked increases in prevalence and burden, but large UIs prohibit definite conclusions. New studies with more detailed case ascertainment should be performed to determine whether these increases are real.

All figures on DALYs, YLDs, and YLLs for the individual disorders are given with UIs, illustrating their accuracy and the need for correct interpretation. As the overall burden of brain disorders was calculated by the authors through summing of the individual disorders, 95% uncertainty intervals could not be directly estimated for the aggregated burden.

Most of the figures are not based on Norwegian data sources alone, but represent extrapolations of data on prevalence, incidence, and mortality from other parts of the world, where data from neighboring and “epidemiologically similar” countries will have a particular impact on Norwegian estimates. They are based on intricate mathematical modeling of disorders that should be appropriate for a certain country, disease, age, and sex group. In DisMod-AT, several methods are used to adjust for change in case definitions, diagnostic methods and health care access over time in the underlying data, but residual bias cannot be excluded. Therefore, the accuracy of the figures for Norway can be contested. If some estimates seem surprising, this should be a stimulus to ensure that all relevant data are made available for GBD and conduct new data collections if baseline data are missing. To our knowledge no other studies give data from other countries or regions that are directly comparable to the data given here.

The term “brain disorders,” as used in this paper, is a convenient but somewhat imprecise designation, as it also encompasses some disorders of nervous tissue outside but closely connected to the brain, such as the spinal cord and a few rare muscle disorders. These conditions are included within the category ‘other neurological disorders’, which also comprises diseases such as Huntington's disease, a typical brain disorder. The population burden of all these disorders is relatively small.

GBD data is lacking for some disorders that that are clearly related to the brain, e.g. some sleep disorders, or to the nervous system, e.g. neuropathies which are only captured as a sequela of diabetes. Some chronic pain conditions (e.g. In the neck or low back, or fibromyalgia, the latter not a diagnosis in GBD) may partly be related to some mental or neurological disorders. Also, since GBD only gives prevalence, incidence, and YLDs for brain and spinal cord injuries, neurotrauma were not included when calculating the total burden. Hence, the figures for the category ‘brain disorders’ are minimum figures.

In the GBD results tool, estimates can be obtained for each sex, various age groups, and each county of Norway. In this study, where the main aim was to show the nationwide overall impact of all brain disorders, it was within the limits of one paper possible to give estimates only for both sexes combined, all ages together, and for the whole country. We encourage researchers who wish to understand in depth only one or a few of these disorders to make use of all data available in GBD.

The GBD data allows for forecasting population health,^40^ based on trends in population age and composition, sociodemographic factors and other drivers of health. Forecasting future health burden of brain disorders is highly relevant for planning health services but is outside the scope of this paper.

### Conclusions

Many patients live with brain disorders for a very long time, requiring treatment, support, and care for years and even decades. Some conditions, such as autism and IDID, affect individuals throughout their entire lives. Several of the most common disorders, including ADHD, migraine and other headaches, alcohol and drug dependency, epilepsy, MS, schizophrenia, anxiety, bipolar disorder, and depression, often appear in childhood, adolescence, or young adulthood. These early-onset disorders can significantly limit workforce participation, resulting in high societal costs through disability pensions, sick-leave (“absenteeism”), and reduced productivity while at work (“presenteeism”). They also impact patients’ personal finances and place considerable strain on their families, employers, and colleagues. Since these disorders often emerge during formative years-when individuals pursue education, form important social connections, and establish partnerships–these brain disorders may have secondary and cumulative effects on later aspects of life including careers, social and romantic relationships, marriage, and child-rearing. Disorders that primarily appear later in life, such as stroke, dementia, PD, and MND, often place a heavy caregiving burden on relatives and strain nursing home resources.

The diverse and highly varied manifestations of brain disorders have historically posted challenges to understanding the overall importance of the brain as a source of health loss. Over the past 34 years, encouraging trends have been observed for some brain disorders such as stroke, AD and dementia, CNS traumas and infections, self-harm, and alcohol use disorder. These improvements reflect improvements in general health -particularly vascular health-as well as targeted disease-specific prevention, treatment, and care. In spite of this, the number of patients and thereby the overall societal burden of stroke and dementia will probably remain high because of the continued aging of the population.^40^^,^^41^ For conditions like MS and CNS cancers, reduction in mortality has resulted in an increased number of patients, leading to an overall higher disease burden. It is concerning that certain disorders, such as PD and drug use disorders, show dramatic increases in prevalence, burden, and mortality. Disappointing is also the lack of significant progress for some of the most burdensome disorders, including anxiety, depression and headache disorders, where no improvements in prevalence and burden have been observed over the last thirty years. This underlines the need to detect modifiable risk factors, to develop better prevention and treatment strategies, and to make the present measures available to all patients who need them. The high burden more than anything else reflects the need to identify preventable risk factors, also illustrated by the fact that the GBD study includes few risk factors for mental disorders.

Our findings should guide health policymakers and leaders in prioritizing resources for healthcare services and research, both applied and basic. When evaluating the costs of such initiatives, it is crucial to also consider the potential economic benefits of reducing the burden of brain disorders. Continued monitoring of temporal trends in prevalence and burden of brain disorders is essential for assessing the impact of changes in the general health of the population, as well as the effects of new, often very costly, treatments and targeted health interventions on brain disorders. Such data are critical in shaping effective strategies and ensuring the efficient allocation of resources.

## Contributors

LJS conceptualized and planned the study, extracted all the data, wrote the first draft, reviewed the manuscript, and approved the final version.

AKSK extracted all the data, independently from LJS, reviewed the manuscript, gave particular input to the methods and discussion part, and approved the final version.

LKL reviewed the manuscript, gave particular input to the data and discussion of mental and substance use disorders, and approved the final version.

HP conceptualized and planned the study, reviewed the manuscript and approved the final version.

NEG conceptualized and planned the study, reviewed the manuscript and approved the final version.

LJS and AKSK both accessed and verified the data.

## Data sharing statement

To download the data used in these analyses, please visit the Global Health Data Exchange GBD 2023 website (http://ghdx.healthdata.org/gbd-2023).

## Declaration of interests

Lars Jacob Stovner: None.

Ann Kristin Skrindo Knudsen: None.

Lars Lien: None.

Henrik Peersen: My involvement in the article is through my paid position as Secretary General of the Norwegian Brain Council.

N.E. Gilhus has received honoraria or financial support outside the scope of this article from Alexion, Amgen, Argenx, Denka, Dianthus, Grifols, Huma, Janssen, Johnson & Johnson, Lundbeck, Medison, Merck, NMD Pharma, Novartis, Roche, Takeda, and UCB. He is also a Board member of the European Myasthenia Association (EuMGA).
