# Supplementary material for: The burden of brain disorders in Norway: an analysis of data from the global burden of disease study 2023
Source: Lancet Reg Health Eur. 2026 Sep 10;70:101857. doi: 10.1016/j.lanepe.2026.101857 (PMC13586571; doi:10.1016/j.lanepe.2026.101857)
Supplement: Supplementary file 1 [file mmc1.docx]

## **Supplementary material**

### Table of contents

[**Supplementary material**	1](#_Toc238024181)

[Table of contents	1](#_Toc238024182)

[**Appendix 1 Data sources**	2](#_Toc238024183)

[**Appendix 2 GATHER**	8](#_Toc238024184)

[References	9](#_Toc238024185)

[**Appendix 3 Supplementary figures**	10](#_Toc238024186)

[Legend to figures	10](#_Toc238024187)

[Figure S1	11](#_Toc238024188)

[Figure S1b	12](#_Toc238024189)

[Figure S2	13](#_Toc238024190)

[Figure S3	14](#_Toc238024191)

[Figure S4	15](#_Toc238024192)

[Figure S5	16](#_Toc238024193)

[Figure S6	17](#_Toc238024194)

[Figure S7	18](#_Toc238024195)

[Figure S9	20](#_Toc238024196)

[Figure S10	21](#_Toc238024197)

## **Appendix 1 Data sources**

From: Global Burden of Disease Study 2023 (GBD 2023) Sources Tool
(https://sources.healthdata.org/collection/sources-2023)

Components: Causes of death and Nonfatal health outcomes

| Disorder | Data type | Citation |
| --- | --- | --- |
| Alzheimer’s disease and other dementias | Scientific paper | Forsaa EB, Larsen JP, Wentzel-Larsen T, Goetz CG, Stebbins GT, Aarsland D, Alves G. A 12-year population-based study of psychosis in Parkinson disease. Arch Neurol. 2010; 67(8): 9961001. |
|  | Scientific paper | Gjøra L, Strand BH, Bergh S, Borza T, Brækhus A, Engedal K, Johannessen A, Kvello-Alme M, Krokstad S, Livingston G, Matthews FE, Myrstad C, Skjellegrind H, Thingstad P, Aakhus E, Aam S, Selbæk G. Current and Future Prevalence Estimates of Mild Cognitive Impairment, Dementia, and Its Subtypes in a Population-Based Sample of People 70 Years and Older in Norway: The HUNT Study. J Alzheimers Dis. 2021; 79(3): 1213-1226. |
|  | Scientific paper | Olsen C, Pedersen I, Bergland A, Enders-Slegers MJ, Jøranson N, Calogiuri G, Ihlebæk C. Differences in quality of life in home-dwelling persons and nursing home residents with dementia - a cross-sectional study. BMC Geriatr. 2016; 16: 137. |
|  | Scientific paper | Diaz E, Kumar BN, Engedal K. Immigrant patients with dementia and memory impairment in primary health care in Norway: a national registry study. Dement Geriatr Cogn Disord. 2015; 39(5-6): 321–31. |
|  | Scientific paper | Engedal K, Haugen PK. The Prevalence of Dementia in a Sample of Elderly Norwegians. Int J Geriatr Psychiatry. 1993; 8(7): 565-70. |
|  |  |  |
| Parkinson’s disease | Scientific paper | Brakedal B, Toker L, Haugarvoll K, Tzoulis C. A nationwide study of the incidence, prevalence and mortality of Parkinson's disease in the Norwegian population. NPJ Parkinsons Dis. 2022; 8(1): 19. |
|  | Scientific paper | Herlofson K, Lie SA, Årsland D, Larsen JP. Mortality and Parkinson disease A community based study. Neurology. 2004; 62(6): 937-42. |
|  | Scientific paper | Bekkelund SI SB, Mellgren SI. Parkinsons sykdom i en nordnorsk befolkningsgruppe. Tidsskr Nor Laegeforen. 1989; 109(5): 561-3. |
|  | Scientific paper | Tandberg E, Larsen JP, Nessler EG, Riise T, Aarli JA. The epidemiology of Parkinson's disease in the county of Rogaland, Norway. Mov Disord. 1995; 10(5): 541-9. |
|  | Scientific paper | Cortese M, Riise T, Engeland A, Ascherio A, Bjørnevik K. Urate and the risk of Parkinson's disease in men and women. Parkinsonism Relat Disord. 2018; 52: 76-82. |
|  | Vital registration | Norwegian Institute of Public Health. Norway Cause of Death Registry 1980-2022. Oslo, Norway: Norwegian Institute of Public Health. |
|  |  |  |
| Idiopathic epilepsy | Scientific paper | Breivik N, Reiher T. Epilepsy in children in the Sunnmøre District. Tidsskr Nor Laegeforen. 2008; 128(18): 2049-51. |
|  | Scientific paper | Brodtkorb E, Sjaastad O. Epilepsy prevalence by individual interview in a Norwegian community. Seizure. 2008; 17(7): 646-50. |
|  | Scientific paper | Aaberg KM, Gunnes N, Bakken IJ, Lund Søraas C, Berntsen A, Magnus P, Lossius MI, Stoltenberg C, Chin R, Surén P. Incidence and Prevalence of Childhood Epilepsy: A Nationwide Cohort Study. Pediatrics. 2017; 139(5). |
|  | Scientific paper | Syvertsen M, Nakken KO, Edland A, Hansen G, Hellum MK, Koht J. Prevalence and etiology of epilepsy in a Norwegian county-A population based study. Epilepsia. 2015; 56(5): 699-706. |
|  | Scientific paper | Waaler PE, Blom BH, Skeidsvoll H, Mykletun A. Prevalence, classification, and severity of epilepsy in children in western Norway. Epilepsia. 2000; 41(7): 802-10. |
|  | Vital registration | Norwegian Institute of Public Health. Norway Cause of Death Registry 1980-2022. Oslo, Norway: Norwegian Institute of Public Health. |
|  |  |  |
| Multiple sclerosis | Scientific paper | Grytten N, Aarseth JH, Lunde HM, Myhr KM. A 60-year follow-up of the incidence and prevalence of multiple sclerosis in Hordaland County, Western Norway. J Neurol Neurosurg Psychiatry. 2015; nan. |
|  | Scientific paper | Larsen JP, Riise T, Nyland H, Kvåle G, Aarli JA. Clustering of multiple sclerosis in the county of Hordaland, Western Norway. Acta Neurol Scand. 1985; 71(5): 390-5. |
|  | Scientific paper | Riise T, Grønning M, Klauber MR, Barrett-Connor E, Nyland H, Albrektsen G. Clustering of residence of multiple sclerosis patients at age 13 to 20 years in Hordaland, Norway. Am J Epidemiol. 1991; 133(9): 932-9. |
|  | Scientific paper | Benjaminsen E, Myhr KM, Grytten N, Alstadhaug KB. Comorbidity in multiple sclerosis patients from Nordland County, Norway - validated data from the Norwegian Patient Registry. Mult Scler Relat Disord. 2021; 48: 102691. |
|  | Scientific paper | Midgard R, Riise T, Kvåle G, Nyland H. Disability and mortality in multiple sclerosis in western Norway. Acta Neurol Scand. 1996; 93(5): 307-14. |
|  | Scientific paper | Midgard R, Riise T, Nyland H. Epidemiologic trends in multiple sclerosis in Møre and Romsdal, Norway: a prevalence/incidence study in a stable population. Neurology. 1991; 41(6): 887-92. |
|  | Scientific paper | Edland A, Nyland H, Riise T, Larsen JP. Epidemiology of multiple sclerosis in the county of Vestfold, eastern Norway: incidence and prevalence calculations. Acta Neurol Scand. 1996; 93(2-3): 104-9. |
|  | Scientific paper | Willumsen JS, Aarseth JH, Myhr KM, Midgard R. High incidence and prevalence of MS in Møre and Romsdal County, Norway, 1950-2018. Neurol Neuroimmunol Neuroinflamm. 2020; 7(3): e713. |
|  | Scientific paper | Simonsen CS, Edland A, Berg-Hansen P, Celius EG. High prevalence and increasing incidence of multiple sclerosis in the Norwegian county of Buskerud. Acta Neurol Scand. 2017; 135(4): 412–8. |
|  | Scientific paper | Ghaderi S, Berg-Hansen P, Bakken IJ, Magnus P, Trogstad L, Håberg SE. Hospitalization following influenza infection and pandemic vaccination in multiple sclerosis patients: a nationwide population-based registry study from Norway. Eur J Epidemiol. 2020; 35(4): 355-362. |
|  | Scientific paper | Grønning M, Riise T, Kvåle G, Nyland H, Larsen JP, Aarli JA. Incidence of multiple sclerosis in Hordaland, western Norway: a fluctuating pattern. Neuroepidemiology. 1991; 10(2): 53-61. |
|  | Scientific paper | Smestad C, Sandvik L, Holmoy T, Harbo HF, Celius EG. Marked differences in prevalence of multiple sclerosis between ethnic groups in Oslo, Norway. J Neurol. 2008; 255(1): 49-55. |
|  | Scientific paper | Lund C, Nakken KO, Edland A, Celius EG. Multiple sclerosis and seizures: incidence and prevalence over 40 years. Acta Neurol Scand. 2014; 130(6): 368-73. |
|  | Scientific paper | Dahl OP, Aarseth JH, Myhr KM, Nyland H, Midgard R. Multiple sclerosis in Nord-Trøndelag County, Norway: a prevalence and incidence study. Acta Neurol Scand. 2004; 109(6): 378-84. |
|  | Scientific paper | Grønlie SA, Myrvoll E, Hansen G, Grønning M, Mellgren SI. Multiple sclerosis in North Norway, and first appearance in an indigenous population. J Neurol. 2000; 247(2): 129-33. |
|  | Scientific paper | Celius EG, Vandvik B. Multiple sclerosis in Oslo, Norway: prevalence on 1 January 1995 and incidence over a 25-year period. Eur J Neurol. 2001; 8(5): 463-9. |
|  | Scientific paper | Benjaminsen E, Olavsen J, Karlberg M, Alstadhaug KB. Multiple sclerosis in the far north--incidence and prevalence in Nordland County, Norway, 1970-2010. BMC Neurol. 2014; 14: 226. |
|  | Scientific paper | Grønning M, Mellgren SI. Multiple sclerosis in the two northernmost counties of Norway. Acta Neurol Scand. 1985; 72(3): 321-7. |
|  | Scientific paper | Vatne A, Mygland A, Ljøstad U. Multiple sclerosis in Vest-Agder County, Norway. Acta Neurol Scand. 2011; 123(6): 396-9. |
|  | Scientific paper | Risberg G, Aarseth JH, Nyland H, Lauer K, Myhr K-M, Midgard R. Prevalence and incidence of multiple sclerosis in Oppland County: a cross-sectional population-based study in a landlocked county of Eastern Norway. Acta Neurol Scand. 2011; 124(4): 250-7. |
|  | Vital registration | Norwegian Institute of Public Health. Norway Cause of Death Registry 1980-2022. Oslo, Norway: Norwegian Institute of Public Health. |
|  |  |  |
| Motor Neuron Disease | Scientific paper | Gundersen MD, Yaseen R, Midgard R. Incidence and clinical features of amyotrophic lateral sclerosis in Møre and Romsdal County, Norway. Neuroepidemiology. 2011; 37(1): 58-63. |
|  | Vital registration | Norwegian Institute of Public Health. Norway Cause of Death Registry 1980-2022. Oslo, Norway: Norwegian Institute of Public Health. |
|  |  |  |
| Headache disorders | Scientific paper | Hagen K, Albretsen C, Vilming ST, Salvesen R, Grønning M, Helde G, Gravdahl G, Zwart JA, Stovner LJ. A 4-year follow-up of patients with medication-overuse headache previously included in a randomized multicentre study. J Headache Pain. 2011; 12(3): 315-22 |
|  | Scientific paper | Hagen K, Zwart J-A, Aamodt AH, Nilsen KB, Bråthen G, Helde G, Stjern M, Tronvik EA, Stovner LJ. A face-to-face interview of participants in HUNT 3: the impact of the screening question on headache prevalence. J Headache Pain. 2008; 9(5): 289–94. |
|  | Scientific paper | Dyb G, Holmen TL, Zwart J-A. Analgesic overuse among adolescents with headache: the Head-HUNT-Youth Study. Neurology. 2006; 66(2): 198-201. |
|  | Scientific paper | Zwart J-A, Dyb G, Hagen K, Svebak S, Stovner LJ, Holmen J. Analgesic overuse among subjects with headache, neck, and low-back pain. Neurology. 2004; 62(9): 1540-4. |
|  | Scientific paper | Tronvik E, Zwart J-A, Hagen K, Dyb G, Holmen TL, Stovner LJ. Association between blood pressure measures and recurrent headache in adolescents: cross-sectional data from the HUNT-Youth study. J Headache Pain. 2011; 12(3): 347-53. |
|  | Scientific paper | Kristiansen HA, Kværner KJ, Akre H, Overland B, Russell MB. Migraine and sleep apnea in the general population. J Headache Pain. 2011; 12(1): 55-61. |
|  | Scientific paper | Krogh AB, Larsson B, Linde M. Prevalence and disability of headache among Norwegian adolescents: A cross-sectional school-based study. Cephalalgia. 2015; 35(13): 1181-91. |
|  | Scientific paper | Hagen K, Zwart JA, Vatten L, Stovner LJ, Bovim G. Prevalence of migraine and non-migrainous headache – head-HUNT, a large population-based study. Cephalalgia. 2000; 20(10): 900-6. |
|  | Scientific paper | Hagen K, Linde M, Steiner TJ, Stovner LJ, Zwart J-A. Risk factors for medication-overuse headache: an 11-year follow-up study. The Nord-Trondelag Health Studies. Pain. 2012; 153(1): 56–61. |
|  | Scientific paper | Zwart J-A, Dyb G, Holmen TL, Stovner LJ, Sand T. The prevalence of migraine and tension-type headaches among adolescents in Norway. The Nord-Trøndelag Health Study (Head-HUNT-Youth), a large population-based epidemiological study. Cephalalgia. 2004; 24(5): 373-9. |
|  | Scientific paper | Linde M, Stovner LJ, Zwart J-A, Hagen K. Time trends in the prevalence of headache disorders. The Nord-Trondelag Health Studies (HUNT 2 and HUNT 3). Cephalalgia. 2011; 31(5): 585-96. |
|  |  |  |
| Other Neurological Disorders | Vital registration | Norwegian Institute of Public Health. Norway Cause of Death Registry 1980-2022. Oslo, Norway: Norwegian Institute of Public Health. |
|  |  |  |
| Stroke | Scientific paper | Sandvei M, Mathiesen E, Vatten L, Müller T, Lindekleiv H, Ingebrigtsen T, Njølstad I, Wilsgaard T, Løchen M, Vik A, Romundstad P. Incidence and mortality of aneurysmal subarachnoid hemorrhage in two Norwegian cohorts, 1984-2007. Neurology. 2011; 77(20): 1833-9. |
|  | Scientific paper | Tveiten A, Ljøstad U, Mygland A, Thomassen L, Pripp AH, Naess H. Intracerebral hemorrhage in southern Norway – a hospital-based incidence study. Eur Neurol. 2012; 67(4): 240-5. |
|  | Scientific paper | Naess H, Gjerde G, Waje-Andreassen U. Ischemic stroke in patients older and younger than 80 years. Acta Neurol Scand. 2014; 129(6): 399–404. |
|  | Scientific paper | Barra M, Labberton AS, Faiz KW, Lindstrom JC, Ronning OM, Viana J, Dahl FA, Rand K. Stroke incidence in the young: evidence from a Norwegian register study. J Neurol. 2019; 266(1): 68-84. |
|  | Scientific paper | Vangen-Lønne AM, Wilsgaard T, Johnsen SH, Carlsson M, Mathiesen EB. Time trends in incidence and case fatality of ischemic stroke: the tromsø study 1977-2010. Stroke. 2015; 46(5): 1173–9. |
|  | Scientific paper | Nawaz B, Eide GE, Fromm A, Oygarden H, Sand KM, Thomassen L, Naess H, Waje-Andreassen U. Young ischaemic stroke incidence and demographic characteristics - The Norwegian stroke in the young study - A three-generation research program. Eur Stroke J. 2019; 4(4): 347-54. |
|  | Administrative | Norwegian Directorate of Health. Norway Patient Register 2008-2012 |
|  |  |  |
| Schizophrenia | Scientific paper | Ekeberg KA, Abebe DS. Mental disorders among young adults of immigrant background: a nationwide register study in Norway. Soc Psychiatry Psychiatr Epidemiol. 2021; 56(6): 953-962. |
|  | Scientific paper | Evensen S, Wisløff T, Lystad JU, Bull H, Ueland T, Falkum E. Prevalence, Employment Rate, and Cost of Schizophrenia in a High-Income Welfare Society: A Population-Based Study Using Comprehensive Health and Welfare Registers. Schizophr Bull. 2016; 42(2): 476–83. |
|  | Scientific paper | Madsen KB, Hohwü L, Zhu JL, Olsen J, Obel C. Social selection in cohort studies and later representation of childhood psychiatric diagnoses: The Danish National Birth Cohort. Scand J Public Health. 2020; 48(2): 207-213. |
|  | Scientific paper | Nesvaag R, Knudsen GP, Bakken IJ, Høye A, Ystrom E, Surén P al, Reneflot A, Stoltenberg C, Reichborn-Kjennerud T. Substance use disorders in schizophrenia, bipolar disorder, and depressive illness: a registry-based study. Soc Psychiatry Psychiatr Epidemiol. 2015; 50(8): 1267. |
|  |  |  |
| Depresive disorders | Scientific paper | Kringlen E, Torgersen S, Cramer V. A Norwegian psychiatric epidemiological study. Am J Psychiatry. 2001; 158(7): 1091-8. |
|  | Scientific paper | Askim Å, Gustad LT, Paulsen J, Reitan SK, Mehl A, Mohus RM, Dewan A, Damås JK, Solligård E, Åsvold BO. Anxiety and Depression Symptoms in a General Population and Future Risk of Bloodstream Infection: The HUNT Study. Psychosom Med. 2018; 80(7): 673-679. |
|  | Scientific paper | Ayuso-Mateos JL, Vázquez-Barquero JL, Dowrick C, Lehtinen V, Dalgard OS, Casey P, Wilkinson C, Lasa L, Page H, Dunn G, Wilkinson G. Depressive disorders in Europe: prevalence figures from the ODIN study. Br J Psychiatry. 2001; 179(4): 308-16. |
|  | Scientific paper | Kringlen E, Torgersen S, Cramer V. Mental illness in a rural area: a Norwegian psychiatric epidemiological study. Soc Psychiatry Psychiatr Epidemiol. 2006; 41(9): 713-9. |
|  | Scientific paper | Dahl AA, Grotmol KS, Hjermstad MJ, Kiserud CE, Loge JH. Norwegian reference data on the Fatigue Questionnaire and the Patient Health Questionnaire-9 and their interrelationship. Ann Gen Psychiatry. 2020; 19: 60. |
|  | Scientific paper | Dahl KE, Calogiuri G, Jönsson B. Perceived oral health and its association with symptoms of psychological distress, oral status and socio-demographic characteristics among elderly in Norway. BMC Oral Health. 2018; 18(1): 93. |
|  | Scientific paper | Sund AM, Larsson B, Wichstrøm L. Prevalence and characteristics of depressive disorders in early adolescents in central Norway. Child Adolesc Psychiatry Ment Health. 2011; 5: 28. |
|  | Scientific paper | Gustavson K, Knudsen AK, Nesvag R, Knudsen GP, Vollset SE, Reichborn-Kjennerud T. Prevalence and stability of mental disorders among young adults: findings from a longitudinal study. BMC Psychiatry. 2018; 18(1): 65. |
|  | Scientific paper | Wichstrøm L, Berg-Nielsen TS, Angold A, Egger HL, Solheim E, Sveen TH. Prevalence of psychiatric disorders in preschoolers. J Child Psychol Psychiatry. 2012; 53(6): 695-705. |
|  | Scientific paper | Heiervang E, Stormark KM, Lundervold AJ, Heimann M, Goodman R, Posserud M-B, Ullebø AK, Plessen KJ, Bjelland I, Lie SA, Gillberg C. Psychiatric disorders in Norwegian 8- to 10-year-olds: an epidemiological survey of prevalence, risk factors, and service use. J Am Acad Child Adolesc Psychiatry. 2007; 46(4): 438-47. |
|  | Scientific paper | von Soest T, Wichstrom L. Secular trends in depressive symptoms among Norwegian adolescents from 1992 to 2010. J Abnorm Child Psychol. 2014; 42(3): 403–15. |
|  | Survey | European Commission (EC), Eurostat. Norway European Health Interview Survey 2015 - Eurostat. Luxembourg City, Luxembourg: Eurostat. |
|  | Survey | World Health Organization (WHO). Norway World Health Survey 2003. Geneva, Switzerland: World Health Organization (WHO), 2005. |
|  | Report | Brunes A. The Association Between Physical Activity, Mental Health, and Personality: The HUNT Study [master's thesis]. [Trondheim, Norway]: Norwegian University of Science and Technology; 2011. |
|  |  |  |
| Bipolar disorder | Scientific paper | Kringlen E, Torgersen S, Cramer V. A Norwegian psychiatric epidemiological study. Am J Psychiatry. 2001; 158(7): 1091-8. |
|  | Scientific paper | Kringlen E, Torgersen S, Cramer V. Mental illness in a rural area: a Norwegian psychiatric epidemiological study. Soc Psychiatry Psychiatr Epidemiol. 2006; 41(9): 713-9. |
|  |  |  |
| Anxiety | Scientific paper | Knudsen AK, Stene-Larsen K, Gustavson K, Hotopf M, Kessler RC, Krokstad S, Skogen JC, Øverland S, Reneflot A. Prevalence of mental disorders, suicidal ideation and suicides in the general population before and during the COVID-19 pandemic in Norway: A population-based repeated cross-sectional analysis. Lancet Reg Health Eur. 2021; 4: 100071 |
|  | Scientific paper | Wichstrøm L, Berg-Nielsen TS, Angold A, Egger HL, Solheim E, Sveen TH. Prevalence of psychiatric disorders in preschoolers. J Child Psychol Psychiatry. 2012; 53(6): 695-705. |
|  | Scientific paper | Sandanger I, Nygård JF, Ingebrigtsen G, Sørensen T, Dalgard OS. Prevalence, incidence and age at onset of psychiatric disorders in Norway. Soc Psychiatry Psychiatr Epidemiol. 1999; 34(11): 570-9. |
|  |  |  |
| Eating disorders | Scientific paper | Götestam KG, Agras WS. General Population-Based Epidemiological Study of Eating Disorders in Norway. Int J Eat Disord. 1995; 18(2): 119-26. |
|  | Scientific paper | Halvorsen I, Andersen A, Heyerdahl S. Good outcome of adolescent onset anorexia nervosa after systematic treatment: intermediate to long-term follow-up of a representative county-sample. Eur Child Adolesc Psychiatry. 2004; 13(5): 295-306. |
|  | Scientific paper | Kjelsås E, Bjørnstrøm C, Götestam KG. Prevalence of eating disorders in female and male adolescents (14-15 years). Eat Behav. 2004; 5(1): 13-25. |
|  | Scientific paper | Heiervang E, Stormark KM, Lundervold AJ, Heimann M, Goodman R, Posserud M-B, Ullebø AK, Plessen KJ, Bjelland I, Lie SA, Gillberg C. Psychiatric disorders in Norwegian 8- to 10-year-olds: an epidemiological survey of prevalence, risk factors, and service use. J Am Acad Child Adolesc Psychiatry. 2007; 46(4): 438-47. |
|  | Scientific paper | Zachrisson HD, Vedul-Kjelsås E, Götestam KG, Mykletun A. Time trends in obesity and eating disorders. Int J Eat Disord. 2008; 41(8): 673-80. |
|  | Vital registration | Norwegian Institute of Public Health. Norway Cause of Death Registry 1980-2022. Oslo, Norway: Norwegian Institute of Public Health. |
|  |  |  |
| ADHD | Scientific paper | Kamai EM, Villanger GD, Nethery RC, Thomsen C, Sakhi AK, Drover SSM, Hoppin JA, Knudsen GP, Reichborn-Kjennerud T, Zeiner P, Overgaard K, Herring AH, Aase H, Engel SM. Gestational Phthalate Exposure and Preschool Attention Deficit Hyperactivity Disorder in Norway. Environ Epidemiol. 2021; 5(4): e161. |
|  | Scientific paper | Wichstrøm L, Berg-Nielsen TS, Angold A, Egger HL, Solheim E, Sveen TH. Prevalence of psychiatric disorders in preschoolers. J Child Psychol Psychiatry. 2012; 53(6): 695-705. |
|  | Scientific paper | Heiervang E, Stormark KM, Lundervold AJ, Heimann M, Goodman R, Posserud M-B, Ullebø AK, Plessen KJ, Bjelland I, Lie SA, Gillberg C. Psychiatric disorders in Norwegian 8- to 10-year-olds: an epidemiological survey of prevalence, risk factors, and service use. J Am Acad Child Adolesc Psychiatry. 2007; 46(4): 438-47. |
|  |  |  |
| Autism spectrum disorder | Scientific paper | Kamai EM, Villanger GD, Nethery RC, Thomsen C, Sakhi AK, Drover SSM, Hoppin JA, Knudsen GP, Reichborn-Kjennerud T, Zeiner P, Overgaard K, Herring AH, Aase H, Engel SM. Gestational Phthalate Exposure and Preschool Attention Deficit Hyperactivity Disorder in Norway. Environ Epidemiol. 2021; 5(4): e161. |
|  | Scientific paper | Wichstrøm L, Berg-Nielsen TS, Angold A, Egger HL, Solheim E, Sveen TH. Prevalence of psychiatric disorders in preschoolers. J Child Psychol Psychiatry. 2012; 53(6): 695-705 |
|  | Scientific paper | Heiervang E, Stormark KM, Lundervold AJ, Heimann M, Goodman R, Posserud M-B, Ullebø AK, Plessen KJ, Bjelland I, Lie SA, Gillberg C. Psychiatric disorders in Norwegian 8- to 10-year-olds: an epidemiological survey of prevalence, risk factors, and service use. J Am Acad Child Adolesc Psychiatry. 2007; 46(4): 438-47. |
|  |  |  |
| Conduct disorder | Scientific paper | Wichstrøm L, Berg-Nielsen TS, Angold A, Egger HL, Solheim E, Sveen TH. Prevalence of psychiatric disorders in preschoolers. J Child Psychol Psychiatry. 2012; 53(6): 695-705. |
|  | Scientific paper | Heiervang E, Stormark KM, Lundervold AJ, Heimann M, Goodman R, Posserud M-B, Ullebø AK, Plessen KJ, Bjelland I, Lie SA, Gillberg C. Psychiatric disorders in Norwegian 8- to 10-year-olds: an epidemiological survey of prevalence, risk factors, and service use. J Am Acad Child Adolesc Psychiatry. 2007; 46(4): 438-47. |
|  |  |  |
| Idiopathic developmental intellectual disability | Scientific paper | Strømme P, Valvatne K. Mental retardation in Norway: prevalence and sub-classification in a cohort of 30037 children born between 1980 and 1985. Acta Paediatr. 1998; 87(3): 291-6. |
|  | Scientific paper | Søndenaa E, Rasmussen K, Nøttestad JA, Lauvrud C. Prevalence of intellectual disabilities in Norway: Domestic variance. J Intellect Disabil Res. 2010; 54(2): 161-7. |
|  |  |  |
| Othe mental disorders |  | Not available |
|  |  |  |
| Self-harm | Administrative | Norway Hospital Discharge Injury Tabulations 2004 as provided by the Global Burden of Disease 2010 Injury Expert Group. [Unpublished]. |
|  | Vital registration | Norwegian Institute of Public Health. Norway Cause of Death Registry 1980-2022. Oslo, Norway: Norwegian Institute of Public Health. |
|  |  |  |
| Brain and CNS cancer | Disease registry | Cancer Registry of Norway. Cancer in Norway 2017. Oslo, Norway: Cancer Registry of Norway, 2018. |
|  | Disease registry | Curado MP, Edwards B, Shin HR, Storm H, Ferlay J, Heanue M and Boyle P, eds (2007). Cancer Incidence in Five Continents, Vol. IX Periodic Data (electronic version). Lyon, IARC. http://ci5.iarc.fr |
|  | Disease registry | Norway Cancer Registry 2008-2012 - CI5. |
|  | Disease registry | Association of Nordic Cancer Registries (ANCR). Norway NORDCAN Cancer Incidence Data Tables, Age-Specific by Countries. Copenhagen, Denmark: Association of Nordic Cancer Registries (ANCR). |
|  | Disease registry | Association of Nordic Cancer Registries (ANCR). Norway NORDCAN Cancer Mortality Data Tables, Age-Specific by Countries. Copenhagen, Denmark: Association of Nordic Cancer Registries (ANCR). |
|  | Disease registry | Norway Cancer Registry 2008-2012 - CI5. |
|  | Modeled data | Institute for Health Metrics and Evaluation (IHME). IHME GBD Cancer Incidence and Mortality Estimates. |
|  | Vital registration | Norwegian Institute of Public Health. Norway Cause of Death Registry 1980-2022. Oslo, Norway: Norwegian Institute of Public Health. |
|  |  |  |
| Alcohol use disorders | Scientific paper | Kringlen E, Torgersen S, Cramer V. A Norwegian psychiatric epidemiological study. Am J Psychiatry. 2001; 158(7): 1091-8. |
|  | Scientific paper | Gustavson K, Knudsen AK, Nesvag R, Knudsen GP, Vollset SE, Reichborn-Kjennerud T. Prevalence and stability of mental disorders among young adults: findings from a longitudinal study. BMC Psychiatry. 2018; 18(1): 65. |
|  | Scientific paper | Elgen I, Bruaroy S, Laegreid LM. Lack of recognition and complexity of foetal alcohol neuroimpairments. Acta Paediatr. 2007; 96(2): 237-41. |
|  | Scientific paper | Kringlen E, Torgersen S, Cramer V. Mental illness in a rural area: a Norwegian psychiatric epidemiological study. Soc Psychiatry Psychiatr Epidemiol. 2006; 41(9): 713-9. |
|  | Disease registry | European Surveillance of Congenital Anomalies (EUROCAT). Norway EUROCAT Prevalence Tables, Individual Registries and Countries. Newtownabbey, Northern Ireland: European Surveillance of Congenital Anomalies (EUROCAT). |
|  | Vital registration | Norwegian Institute of Public Health. Norway Cause of Death Registry 1980-2022. Oslo, Norway: Norwegian Institute of Public Health. |
|  |  |  |
| Drug use disorders | Scientific paper | Haarr D, Nessa J. [Treatment of opiate-dependent patients in a general practice]. Tidsskr Nor Laegeforen. 2007; 127(13): 1770-2. |
|  | Scientific paper | Shi Y, Lenzi M, An R. Cannabis Liberalization and Adolescent Cannabis Use: A Cross-National Study in 38 Countries. PLoS One. 2015; 10(11): e0143562. |
|  | Scientific paper | Rossow I, Hawton K, Ystgaard M. Cannabis use and deliberate self-harm in adolescence: a comparative analysis of associations in England and Norway. Arch Suicide Res. 2009; 13(4): 340–8. |
|  | Scientific paper | Eskild A, Magnus P, Samuelsen SO, Sohlberg C, Kittelsen P. Differences in mortality rates and causes of death between HIV positive and HIV negative intravenous drug users. Int J Epidemiol. 1993; 22(2): 315-20. |
|  | Scientific paper | Ødegård E, Amundsen EJ, Kielland KB. Fatal overdoses and deaths by other causes in a cohort of Norwegian drug abusers – a competing risk approach. Drug Alcohol Depend. 2007; 89(2-3): 176-82. |
|  | Scientific paper | Mathers BM, Degenhardt L, Bucello C, Lemon J, Wiessing L, Hickman M. Mortality among people who inject drugs: a systematic review and meta-analysis. Bull World Health Organ. 2013; 91(2): 102-23. |
|  | Scientific paper | Clausen T, Anchersen K, Waal H. Mortality prior to, during and after opioid maintenance treatment (OMT): a national prospective cross-registry study. Drug Alcohol Depend. 2008; 94(1-3): 151-7. |
|  | Scientific paper | Rossow I. Suicide among drug addicts in Norway. Addiction. 1994; 89(12): 1667-73. |
|  | Survey | World Health Organization Regional Office for Europe (EURO-WHO). Health Behaviour in School-aged Children: WHO Collaborative Cross-National survey/study (HBSC) 2009-2010. |
|  | Vital registration | Norwegian Institute of Public Health. Norway Cause of Death Registry 1980-2022. Oslo, Norway: Norwegian Institute of Public Health. |
|  | Report | European School Survey Project on Alcohol and Other Drugs (ESPAD), Pompidou Group, Council of Europe, Swedish Council for Information on Alcohol and Other Drugs (CAN). ESPAD Report 2003: Alcohol and Other Drug Use Among Students in 35 European Countries. Stockholm, Sweden: Swedish Council for Information on Alcohol and Other Drugs (CAN), 2004. |
|  | Report | European Monitoring Centre for Drugs and Drug Addiction (EMCDDA). ESPAD Report 2015: Results from the European School Survey Project on Alcohol and Other Drugs. Lisbon, Portugal: European School Survey Project on Alcohol and Other Drugs (ESPAD), 2016. |
|  | Report | European Monitoring Centre for Drugs and Drug Addiction (EMCDDA). European Monitoring Centre for Drugs and Drug Addiction Statistical Bulletin 2008. Lisbon, Portugal: European Monitoring Centre for Drugs and Drug Addiction (EMCDDA). |
|  | Report | European Monitoring Centre for Drugs and Drug Addiction (EMCDDA). European Monitoring Centre for Drugs and Drug Addiction Statistical Bulletin 2013. Lisbon, Portugal: European Monitoring Centre for Drugs and Drug Addiction (EMCDDA). |
|  | Report | European Monitoring Centre for Drugs and Drug Addiction (EMCDDA). European Monitoring Centre for Drugs and Drug Addiction Statistical Bulletin 2015. Lisbon, Portugal: European Monitoring Centre for Drugs and Drug Addiction (EMCDDA). |
|  | Report | European Monitoring Centre for Drugs and Drug Addiction (EMCDDA). European Monitoring Centre for Drugs and Drug Addiction Statistical Bulletin 2016. Lisbon, Portugal: European Monitoring Centre for Drugs and Drug Addiction (EMCDDA), 2016. |
|  | Report | European Monitoring Centre for Drugs and Drug Addiction (EMCDDA). European Monitoring Centre for Drugs and Drug Addiction Statistical Bulletin 2018. Lisbon, Portugal: European Monitoring Centre for Drugs and Drug Addiction (EMCDDA). |
|  |  |  |
| Traumatic brain injury |  | References not avalable |
| Spinal cord injury |  | References not available |
|  |  |  |
| Meningitis | Administrative | Norwegian Directorate of Health. Norway Patient Register 2008-2012 |
|  | Vital registration | Norwegian Institute of Public Health. Norway Cause of Death Registry 1980-2022. Oslo, Norway: Norwegian Institute of Public Health. |
| Encephalitis | Administrative | Norwegian Directorate of Health. Norway Patient Register 2008-2012 |
|  | Vital registration | Norwegian Institute of Public Health. Norway Cause of Death Registry 1980-2022. Oslo, Norway: Norwegian Institute of Public Health. |

## **Appendix 2 GATHER**

**Guidelines for Accurate and Transparent Health Estimates Reporting: the GATHER statement^1^**

|  | **Objectives and funding** | Section or other source | Page |
| --- | --- | --- | --- |
| 1 | Define the indicator(s), populations (including age, sex, and geographic entities), and time period(s) for which estimates were made. | Research in context  Introduction (2nd paragraph)  Methods | 3  4  5 |
| 2 | List the funding sources for the work. |  | 2 |
|  | **Data inputs** |  |  |
|  | *For all data inputs from multiple sources that are synthesised as part of the study:* |  |  |
| 3 | Describe how the data were identified and how the data were accessed. | Research in Context  Methods  Data availability statement in the paper. | 3  5  12 |
| 4 | Specify the inclusion and exclusion criteria. Identify all ad-hoc exclusions | Research in Context  Methods  Appendix 1 of reference^2^, section 1.5, and Data input section and sections on inclusion and exclusion criteria for each disorder | 3  5  Reference found at p 13 |
| 5 | Provide information about all included data sources and their main characteristics. For each data source used, report reference information or contact name/institution, population represented, data collection method, year(s) of data collection, sex and age range, diagnostic criteria or measurement method, and sample size, as relevant. | See Appendix 1, and Appendix 2 Data sources for each disorder, of reference^2^  Norwegian data sources found in Supplementary materials | Reference found at p 13  Suppl., p. 3-8 |
| 6 | Identify and describe any categories of input data that have potentially important biases (eg, based on characteristics listed in item 5). | See Appendix 1 of reference^2^, general section on adjustment for biases, and similar section under each disorder | Reference found at p 13 |
|  | *For data inputs that contribute to the analysis but were not synthesised as part of the study:* |  |  |
| 7 | Describe and give sources for any other data inputs. | See Appendix 1 of reference^2^, and Appendix 2 Data sources for each disorder, of reference^2^ | Reference found at p 13 |
| 8 | Provide all data inputs in a file format from which data can be efficiently extracted (eg, a spreadsheet rather than a PDF), including all relevant meta-data listed in item 5. For any data inputs that cannot be shared because of ethical or legal reasons, such as third-party ownership, provide a contact name or the name of the institution that retains the right to the data. | Data are extracted from the GBD interactive website GBD Results, as described in the Research in Context and in Methods. | 3, 5,6 |
|  | **Data analysis** |  |  |
| 9 | Provide a conceptual overview of the data analysis method. A diagram may be helpful. | Methods  See Methods part, and Appendix 1, Methods for each disorder of reference^2^ | 5,6  Reference found at p 13 |
| 10 | Provide a detailed description of all steps of the analysis, including mathematical formulae. This description should cover, as relevant, data cleaning, data pre-processing, data adjustments and weighting of data sources, and mathematical or statistical model(s). | Methods  See Methods part, and Appendix 1, Methods for each disorder, of reference^2^ . | 5, 6  Reference found at p 13 |
| 11 | Describe how candidate models were evaluated and how the final model(s) were selected. | See Methods part, and Appendix 1, Methods for each disorder, of reference^2^ . | Reference found at p 13 |
| 12 | Provide the results of an evaluation of model performance, if done, as well as the results of any relevant sensitivity analysis. | See Methods part, and Appendix 1, Methods for each disorder, of reference^2^. | Reference found at p 13 |
| 13 | Describe methods of calculating uncertainty of the estimates. State which sources of uncertainty were, and were not, accounted for in the uncertainty analysis. | See Appendix 1, section ‘Calculating uncertainty’ Page 17, and in Methods for each disorder, of reference^2^, | Reference found at p 13 |
| 14 | State how analytical or statistical source code used to generate estimates can be accessed | See Appendix 1, Section ‘2.5.3 MR-BRT features’ (page 40-41), of reference^2^. | Reference found at p 13 |
|  | **Results and discussion** |  |  |
| 15 | Provide published estimates in a file format from which data can be efficiently extracted | Tables 1-5,  and Appendix 2 data sources in supplementary materials | Suppl. p 3-8 |
| 16 | Report a quantitative measure of the uncertainty of the estimates (eg, uncertainty intervals) | Uncertainty intervals for all variables reported in Table 1-5 | 6 |
| 17 | Interpret results in light of existing evidence. If updating a previous set of estimates, describe the reasons for changes in estimates. | See Discussion | 8-10 |
| 18 | Discuss limitations of the estimates. Include a discussion of any modelling assumptions or data limitations that aff ect interpretation of the estimate | See Discussion. | 10 |

### References

1.	Stevens GA, Alkema L, Black RE, et al. Guidelines for Accurate and Transparent Health Estimates Reporting: the GATHER statement. *Lancet* 2016; **388**(10062): e19–e23.

2.	Disease GBD, Injury, Risk Factor C. Burden of 375 diseases and injuries, risk-attributable burden of 88 risk factors, and healthy life expectancy in 204 countries and territories, including 660 subnational locations, 1990-2023: a systematic analysis for the Global Burden of Disease Study 2023. *Lancet* 2025; **406**(10513): 1873–922.

## **Appendix 3 Supplementary figures**

## Legend to figures

Figure S1: Prevalence (%) of different brain disorders.
Abbreviations: See Table 1-4.

Figure S1b: Prevalence (%) of different brain disorders excluding Headache.
Abbreviations: See Table 1-4.

Figure S2: Incidence of different brain disorders (per 100 000). Abbreviations: See Table 1-4

Figure S3: Disabilily-Adjusted Life Years (DALYs) of different brain disorders as % of all disorders.	Abbreviations: See Table 1-4.

Figure S4: Years Lived with Disability (YLDs) of different brain disorders as % of all disorders.
Abbreviations: See Table 1-4.

Figure S5: Years of Life Lost (YLLs) of different brain disorders as % of all disorders.
Abbreviations: See Table 1-4.

Figure S6: Percent change in prevalence of different brain disorders between 1990 and 2023.
Abbreviations: See Table 1-4.
*: Uncertainty Interval (UI) not including 0

Figure S7: Percent change in incidence of different brain disorders between 1990 and 2023.
Abbreviations: See Table 1-4.
*: Uncertainty Interval (UI) not including 0

Figure S8: Percent change in DALY rates of different brain disorders between 1990 and 2023.
Abbreviations: See Table 1-4.
*: Uncertainty Interval (UI) not including 0

Figure S9: Percent change in YLD rates of different brain disorders between 1990 and 2023.
Abbreviations: See Table 1-4.
*: Uncertainty Interval (UI) not including 0

Figure S10: Percent change in YLL rates of different brain disorders between 1990 and 2023.
Abbreviations: See Table 1-4.
*: Uncertainty Interval (UI) not including 0

## Figure S1

## Figure S1b

## Figure S2

## Figure S3

## Figure S4

## Figure S5

## Figure S6

## Figure S7

Figure S8

## Figure S9

## Figure S10
